# Subtype-independent near full-length HIV-1 genome sequencing and assembly to be used in large molecular epidemiological studies and clinical management

**DOI:** 10.7448/IAS.18.1.20035

**Published:** 2015-06-25

**Authors:** Sebastian Grossmann, Piotr Nowak, Ujjwal Neogi

**Affiliations:** 1 Division of Clinical Microbiology, Department of Laboratory Medicine, Karolinska Institutet, Stockholm, Sweden; 2 Institute for Pharmacy and Molecular Biotechnology, Heidelberg University, Heidelberg, Germany; 3 Department of Medicine Huddinge, Karolinska Institutet, Karolinska University Hospital, Stockholm, Sweden

**Keywords:** HIV-1 NFLG sequencing, subtype independent, molecular epidemiology

## Abstract

**Introduction:**

HIV-1 near full-length genome (HIV-NFLG) sequencing from plasma is an attractive multidimensional tool to apply in large-scale population-based molecular epidemiological studies. It also enables genotypic resistance testing (GRT) for all drug target sites allowing effective intervention strategies for control and prevention in high-risk population groups. Thus, the main objective of this study was to develop a simplified subtype-independent, cost- and labour-efficient HIV-NFLG protocol that can be used in clinical management as well as in molecular epidemiological studies.

**Methods:**

Plasma samples (*n*=30) were obtained from HIV-1B (*n*=10), HIV-1C (*n*=10), CRF01_AE (*n*=5) and CRF01_AG (*n*=5) infected individuals with minimum viral load >1120 copies/ml. The amplification was performed with two large amplicons of 5.5 kb and 3.7 kb, sequenced with 17 primers to obtain HIV-NFLG. GRT was validated against ViroSeq™ HIV-1 Genotyping System.

**Results:**

After excluding four plasma samples with low-quality RNA, a total of 26 samples were attempted. Among them, NFLG was obtained from 24 (92%) samples with the lowest viral load being 3000 copies/ml. High (>99%) concordance was observed between HIV-NFLG and ViroSeq™ when determining the drug resistance mutations (DRMs). The N384I connection mutation was additionally detected by NFLG in two samples.

**Conclusions:**

Our high efficiency subtype-independent HIV-NFLG is a simple and promising approach to be used in large-scale molecular epidemiological studies. It will facilitate the understanding of the HIV-1 pandemic population dynamics and outline effective intervention strategies. Furthermore, it can potentially be applicable in clinical management of drug resistance by evaluating DRMs against all available antiretrovirals in a single assay.

## Introduction

The increasing HIV diversity and evolution of circulating and unique recombinants forms (CRFs and URFs) pose a major threat to accurate identification of the circulating HIV strains in an epidemic. This has an impact on development, implementation and maintenance of effective preventive and treatment intervention strategies including vaccine development [1,2]. Due to high genetic heterogeneity, methods for universal amplification and sequencing of diverse HIV-1 subtypes remain inadequate [3]. Regularly, a smaller gene portion is used to characterize the subtype, which limits accurate identification of the HIV-1 strains, especially for the recombinant forms. Our earlier study identified a significantly higher prevalence of recombinant forms when two or more genes were used for subtyping [4]. Moreover, using a smaller portion of the genome limits the identification of epidemiological signatures and recombination hot spots [5], adaptations in a host population and immune recognition at whole genome level [6,7].

In current clinical practice of the genotypic resistance testing (GRT), most of the commercial or in-house assays provide information about the HIV-1 drug resistance mutations (DRMs) restricted to full-length protease (PR) (1–99) and partial reverse transcriptase (RT) (1–335). However, a growing body of scientific evidence suggests the role of connection domain mutations, for example, N348I [8], in conferring resistance to reverse transcriptase inhibitors (RTIs). Furthermore, distal non-drug target HIV-1 Gag mutations have been described to confer strong resistance to protease inhibitor (PI) [9,10].

Several studies have attempted to develop protocols for HIV-1 near full-length genome (HIV-NFLG) sequencing. However, most of the studies were restricted to sequencing of single subtypes [2,11] or identification of CRFs [12,13]. Moreover, genetic material was mainly derived from cultured cells or as proviral DNA from blood mononuclear cells [14–16]. Data directly from plasma samples are scarce, albeit they are routinely used in clinical drug resistance testing [2,17] and reflect the most recent viral population in the host [18].

Among the HIV-1 groups, the M-group dominates in global infections. Among the M-group subtypes, HIV-1 subtype C (HIV-1C); HIV-1A (A1 and A2); HIV-1B; CRF02_AG and CRF01_AE account for nearly 84% of the global infections [19]. In our recent study from the Swedish InfCare cohort based on the *pol* gene, we identified a highly diverse HIV-1 epidemic dominated by HIV-1B (47%), HIV-1C (18%) and CRF01_AE (12%) [20].

The aim of the present study was to develop a simple, cost and labour-efficient protocol for HIV-NFLG sequencing for diverse HIV-1 subtypes. This protocol could be used routinely in large-scale population-based molecular epidemiological studies. Additionally, this protocol can also be implemented for extended drug resistance genotyping with full-length Gag for predictors of PI-DRMs, full-length PR and RT, Integrase (IN) for Integrase Inhibitor (INI) as well as genotypic co-receptor tropism testing for co-receptor antagonists. Here, we amplified, sequenced and assembled HIV-1B, HIV-1C, CRF01_AE and CRF02_AG NFLG. Therefore, this protocol might potentially serve as a single tool for both epidemiological and clinical studies, independent of HIV-1 subtypes.

## Methods

### Ethical consideration

Ethical permissions were obtained from the Regional Ethics Committee Stockholm (Dnr: 2006/1367-31/4). The patient information was anonymized and de-linked prior to analysis. Single peripheral blood samples were obtained during the routine viral load testing and GRT using ViroSeq™ HIV-1 Genotyping System (Celera Diagnostics, Alameda, CA, USA).

### Patients material and RNA extraction

The patients were followed-up at the Infectious Disease Clinic at Karolinska University Hospital, Stockholm, Sweden, as part of a large cohort, InfCare HIV [20]. Based on *pol* gene subtyping, a total of 30 samples from four different HIV-1 subtypes (HIV-1B (*n*=10), HIV-1C (*n*=10), 01_AE (*n*=5) and 02_AG (*n*=5)) were attempted for HIV-NFLG sequencing. Viral RNA was extracted using QIAamp viral RNA extraction kit (Qiagen, Hilden, Germany) from 140 µl of plasma. RNA was quantified and purity checked using NanoDrop (Thermo Scientific, DE, USA) and stored at −80°C until used. Among the 30 samples, four samples gave A260/A280 and 260/A230 ratio below one and were therefore excluded from the study.

### Primer and cDNA synthesis

**Figure 1:**
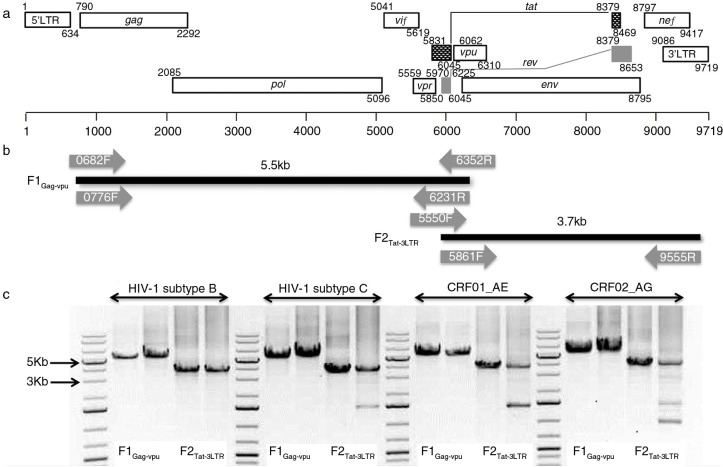
Amplification strategy of HIV-1 NFLG. (a) HIV-1 gene map in HXB2 [GenBank:K03455] co-ordinates Adapted from Los Alamos Database (www.hiv.lanl.gov). (b) The locations of all primers used for cDNA synthesis, nested and semi-nested PCR are shown. The 8.8 kb genome is amplified into two fragments: fragment 1, 5.5 kb (gag-vpu) and fragment 2, 3.7 kb (vif-3′-LTR) with 400 bp overlap. (c) Representative amplification of all four subtypes is presented.

The near full-length genome from the samples was amplified in two fragments using nested and semi-nested polymerase chain reactions (PCR) with seven sets of primers numbered as HXB2 [GenBank:K03455] co-ordinates (Figure 1a and Table 1). First-strand cDNA synthesis was performed using the SuperScript^®^ III RT enzyme (Invitrogen, Life Technologies, MA, USA). For fragment 1 Gag-Vpu (herein F1_Gag-Vpu_), cDNA was synthesized with the primer 6352R (10 pmol) and for fragment 2, Tat-3LTR (herein F2_Tat-3LTR_), an oligo (dT)_18_ primer (50 pmol) (Thermo Scientific) was used. A first master mix of 5 µl extracted viral RNA was combined with 1 µl of 10 mM dNTP mix, 1 µl of 6352R or oligo(dT)_18_ primer and 5 µl of PCR grade water. The reaction mix was heated to 65°C for five minutes and immediately placed on ice for two minutes. A second master mix consisting of 4 µl of 5× First-Strand Buffer (250 mM Tris-HCl, 375 mM KCl, 15 mM MgCl_2_), 1 µl 0.1 M DTT, 1 µl of RiboLock RNase inhibitor (40 U/µl; Thermo Scientific) and 2 µl of SuperScript-III RT (200 U/µl) was subsequently added to the first mastermix. The final 20 µl reaction mix was then incubated at 25°C for five minutes followed by 55°C for one hour and finally 70°C for 10 minutes to terminate the reaction.

**Table 1:** Primers used in this study

Primer_ID	Seq (5′→3′)	HXB2 position	Ref.
Amplification primers			
F1_Gag-Vpu_			
0682F	TCTCTCGACGCAGGACTCGGCTTGCTG	0682→0708	[4,21]
0776F	CTAGAAGGAGAGAGAGATGGGTGCGAG	0776→0800	[4]
6352R	GGTACCCCATAATAGACTGTRACCCACAA	6352→6324	[17]
6231R	CTCTCATTGCCACTGTCTTCTGCTC	6231→6207	[17]
F2_Vif-3LTR_			
5550F	AGARGAYAGATGGAACAAGCCCCAG	5550→5574	[17]
5861F	TGGAAGCATCCRGGAAGTCAGCCT	5861→5884	[17]
9555R	TCTACCTAGAGAGACCCAGTACA	9555→9533	Present study
Sequencing primers			
F1_Gag-Vpu_			
0776F	CTAGAAGGAGAGAGAGATGGGTGCGAG	0776→0800	[4]
1231F	TCACCTAGAACTTTRAATGCATGGG	1231→1255	Present study
1817F	TAGAAGAAATGATGACAG	1817→1834	[22]
2586F	AAGCCAGGAATGGATGGCCCA	2586→2606	[23]
2713R	GGATTTTCAGGCCCAATTTTTG	2713→2692	Present study
3885R	CTGCTCCATCTACATAGAA	3885→3867	[24]
4350R	CACAGCTAGCTACTATTTCTTTTGC	4350→4326	[24]
4900F	GGGTTTATTACAGGGACAGCAGAG	4900→4923	[25]
5066R	ATCATCACCTGCCATCTGTTTTCCAT	5066→5041	[26]
6231R	CTCTCATTGCCACTGTCTTCTGCTC	6231→6207	[17]
F2_Vif-3LTR_			
5861F	TGGAAGCATCCRGGAAGTCAGCCT	5861→5884	[17]
6559F	GGGATCAAAGCCTAAAGCCATGTGTAA	6559→6585	[22]
7002F	TTRTTAAATGGTAGTATAGC	7002→7021	Present study
7373R	GAAAAATTCTCCTCYACAATTAAA	7373→7350	Present study
7761F	GTGGGAATAGGAGCTGTGTTCCTTGGG	7761→7787	[22]
8445R	CTCTCTCTCCACCTTCTTCTTC	8445→8424	[22]
9555R	TCTACCTAGAGAGACCCAGTACA	9555→9533	Present study

Source of the primers are given. The primer IDs are changed from the original for the ease of identification. The numbering is as per HXB2 (K03455).

### NFLG PCR and sequencing

All PCR reactions were performed with high fidelity KAPA HiFi HotStart ReadyMix (2×) (KAPA Biosystem, MA, USA) with 15 pmol of each primer in 50 µl total volume. For F1_Gag-Vpu_, the first round PCR was performed with 0682F and 6352R primers and was followed by the second round nested with 0776F and 6231R primers, which yielded an amplicon of approximately 5.5 kb (Figure 1b). The condition for both PCRs was as follows: initial denaturation at 95°C for five minutes followed by 30 cycles of 98°C for 20 sec, 65°C for 15 sec and 72°C for three minutes and final extension at 72°C for five minutes. The F2_Tat-3LTR_ fragment was amplified in a semi-nested manner with the first and second round forward primers 5550F and 5831F, respectively, with a common reverse primer 9555R, yielding a 3.7 kb amplicon (Figure 1b). The condition for both the PCRs was as follows: initial denaturation at 95°C for five minutes followed by 30 cycles of 98°C for 20 sec, 65°C for 15 sec and 72°C for two minutes and final extension at 72°C for five minutes. The PCR amplicon was gel purified using QIAamp Gel Extraction Kit (Qiagen). Sequencing was performed with a set of 17 primers (Table 1). Representative PCR amplification of all the four subtypes was presented in Figure 1c.

### Sequence assembly, visualization and quality control

The sequencing primers were designed in such a way that there will be 100 bases overlapping with 800 bp sequencing read (Table 1). The sequencing was performed with the Applied Biosystems^®^ 3730xl DNA Analyzer (Life Technologies, CA, USA). The sequences were auto-clipped with a quality score ≥10. CAP3 Sequence Assembly Program with default parameter was used to assemble the final sequence from all available contigs [27]. Due to high genetic variability, only the major peak was considered in the consensus sequence. Single-base frame shifts due to sequencing errors were curated manually after observation of the chromatogram or alignment with the subtype-specific consensus sequences in ClustalX2 [28]. A multiple sequence alignment against the HXB2 reference genome was generated and analyzed with an in-house Perl script (Supplementary file 1) that recognizes the nucleotide changes from the HXB2 sequence [GenBank:K03455] and creates a corresponding number code as per HXB2 co-ordinates. The resulting matrix was plotted using the TraMineR package [29] in R version 3.1.2 [30]. Sequence quality control was performed using Quality Control Tool available in Los Alamos database (www.hiv.lanl.gov/) and submitted to GenBank under accession nos. KP411822 to KP411845.

### Subtyping, drug resistance and co-receptor tropism

HIV-1 subtyping was performed with COMET-HIV, which uses adaptive context-based modelling for ultrafast HIV-1 subtype identification [31]. A maximum likelihood phylogenetic tree with reference sequences was generated in MEGA6 software [32] using a General Time Reversible (GTR) model with inverse gamma distribution. The reference sequences were downloaded from Los Alamos database (www.hiv.lanl.gov). GRT was performed with whole *pol* gene that provides DRM profile of PR, RT and IN. The results were compared with the ViroSeq™ HIV-1 Genotyping System (Life Technologies), which provide DRM profile of full PR and first 335 amino acids of RT. Co-receptor tropism analysis was performed using Geno2pheno_[co-receptor]_ with 10% false-positive rate [33].

## Results

The patients (*n*=26) demographic and clinical characteristics along with the amplification proficiency are given in Table 2.

**Table 2:** Demographic and clinical parameters and amplification of HIV-1 fragment of the study samples

PID	Age	Sex	Viral load^a^	CD4^a^	Therapy	Country of transmission	Route of transmission^a^	F1_Gag-Vpu_	F2_vif-3LTR_
SE600001	51	Male	1,905,461	4	Naïve	Sweden	IVDU	+	+
SE600012	39	Male	707,946	190	Naïve	Sweden	MSM	+	+
SE600023	41	Male	194,984	150	Naïve	Sweden	Heterosexual	+	+
SE600034	52	Male	3,467,369	220	Naïve	Sweden	MSM	+	+
SE600035	44	Female	309,030	260	Naïve	Sweden	IVDU	+	+
SE600046	50	Male	6,606,934	9	Naïve	NA	MSM	+	+
SE600057	45	Male	50,400	180	Naïve	Sweden	Heterosexual	+	+
SE600063	45	Female	7600	316	Naïve	Sweden	Heterosexual	+	+
SE600068	40	Female	1300	421	Naïve	Sweden	NA	−	+
SE600099	27	Female	1120	510	Naïve	Sweden	Heterosexual	+	−
SE600108	35	Male	150,000	270	Naïve	Sweden	IVDU	+	+
SE600119	39	Male	158,000	184	Naïve	Ethiopia	Heterosexual	+	+
SE600210	42	Male	338,844	133	Naïve	Ethiopia	Heterosexual	+	+
SE600311	58	Male	3990	110	Experience	Ethiopia	Heterosexual	+	+
SE600412	58	Male	18,400	300	Naïve	Eritrea	Heterosexual	+	+
SE600213	25	Female	110,000	169	Naïve	Ethiopia	Heterosexual	+	+
SE600314	40	Female	219,000	400	Naïve	Zimbabwe	Heterosexual	+	+
SE600415	30	Male	575,000	125	Naïve	Eritrea	Heterosexual	+	+
SE600122	38	Female	6580	460	Naïve	Eretria	Heterosexual	+	+
SE600516	37	Female	1,000,000	304	Naïve	Sweden	Heterosexual	+	+
SE601017	34	Female	2,300,000	16	Naïve	Thailand	Heterosexual	+	+
SE601018	35	Female	260,000	350	Naïve	Thailand	Heterosexual	+	+
SE601021	40	Female	40,800	249	Naïve	Thailand	NA	+	+
SE602019	41	Female	129,000	400	Naïve	Senegal	Blood transfusion	+	+
SE602020	24	Female	162,000	90	Experience	Cameroon	Heterosexual	+	+
SE602024	35	Female	3060	360	Naïve	Cameroon	Heterosexual	+	+

a Viral load in copies/ml, CD4 in cells/mm^3^. IVDU: intravenous drug user; MSM: men who have sex with men; NA: not available.

Among the 26 samples with viral loads ranging from 1120 to 6,606,934 copies/ml, both PCR fragments were amplified and sequenced from 24 samples (efficiency 92%). In two samples with viral loads below 2000 copies/ml (1300 and 1120 copies/ml respectively), either one of the fragments was not amplified. Amplification was successful in all remaining samples with the lowest viral load being 3060 copies/ml.

For F1_Gag-Vpu_, 10 primers were used which gave complete coverage from the HXB2 positions 790 to 6200. The F2_Tat-3LTR_ amplicon is considerably shorter (~3.7 kb) but still required seven sequencing primers. This was due to the high intra-individual HIV-1 genetic diversity and the presence of poly-A stretches in the region as poly-A stretches can abruptly terminate the sequencing read.

**Figure 2:**
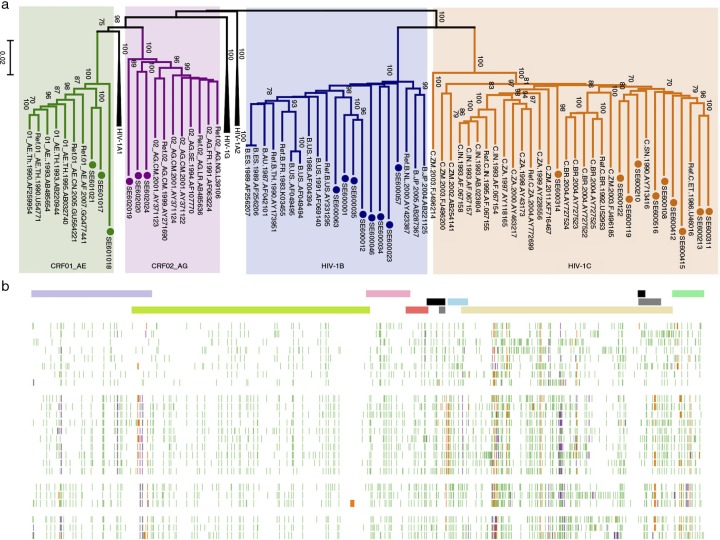
Phylogenetic and variability analysis of sequenced Swedish HIV-1 strains. (a) Maximum likelihood phylogenetic tree with reference HIV-1 sequences downloaded from Los Alamos Database. Four subtypes are indicated: HIV-1B (dark blue), HIV-1C (orange), CRF01_AG (green) and CRF02_AG (purple). The Swedish strains are indicated with filled circle with a respective colour. (b) Genetic diversity of HIV-1 subtypes: all the 24 HIV-1 genomes were aligned with reference to HXB2 [GenBank:K03455]. For each sequence, every nucleotide differing from the reference HXB2 strain (mutation) is shown as a green line, an insertion is shown in orange, and a deletion is shown in purple. The top panel shows the open-reading frame of HIV-1 genes: *gag* (violet), *pol* (lemon green), *vif* (pink), *vpr* (light red), *tat exon 1 and 2* (black), *rev exon 1 and 2* (grey), *vpu* (cyan), env (light yellow) and nef (green).

The phylogenetic analysis identified the same subtype as identified by the *pol* region with 10 as HIV-1C, 8 as HIV-1B and 3 each as 01_AE and 02_AG (Figure 2a). The sequence variability of the 24 samples compared to HXB2 sequence is presented in Figure 2b. This indicates higher sequence variability in the *env* region and the subtype-specific signatures over the genome specifically in the Gag-p6 region.

DRM analysis based on the ViroSeq™ HIV-1 Genotyping System and the current HIV-NFLG assay is presented in Table 3. It should be noted that the HIV-NFLG and the ViroSeq™ showed >99% concordance at 71 DRM positions (PR: 33 positions and RT: 38, excluding N348I, which is not detected by ViroSeq™) in 24 samples (total codon analysis 1704 and three mismatch). In two samples, ViroSeq™ identified PI mutation L10IL (SE600314) and RTI mutations Y318YF (SE602020) in contradiction to the current assay. On the contrary, the V11I mutation was detected by NFLG in one sample (SE600057) but not by ViroSeq™. In two samples, NFLG identified additional N348I mutations due to an extended genomic coverage. Moreover, the current assay potentially can identify the INI-DRMs. The co-receptor tropism identified 18 CCR5-tropic viruses and six as CXCR4-tropic virus (Table 3).

**Table 3:** Comparative drug resistance analysis of current protocol and ViroSeq™ genotypic resistance testing

	ViroSeq genotypic system				Whole genome sequencing						
		
PID	PI major	PI minor	NRTI-DRM	NNRTI-DRM	PI major	PI minor	NRTI-DRM	NNRTI-DRM	INT-DRM-major	INT-DRM-accessory	Co-receptor
SE600001	None	None	None	None	None	None	None	None	None	None	CCR5
SE600012	None	None	None	None	None	None	None	None	None	None	CXCR4
SE600023	None	None	T215S	None	None	None	T215S	None	None	None	CCR5
SE600034	None	None	None	V90I	None	None	None	V90I	None	None	CCR5
SE600035	None	None	None	None	None	None	None	None	None	None	CCR5
SE600046	None	None	None	None	None	None	None	None	None	None	CXCR4
SE600057	None	None	None	None	None	**V11I**	None	None	None	E157Q	CCR5
SE600063	None	None	None	None	None	None	None	None	None	None	CXCR4
SE600108	None	None	None	None	None	None	None	**N348I**	None	None	CCR5
SE600119	None	None	None	None	None	None	None	None	None	None	CCR5
SE600210	None	None	None	None	None	None	None	None	None	None	CCR5
SE600311	None	None	None	V90I	None	None	None	V90I	None	None	CCR5
SE600412	None	None	None	None	None	None	None	None	None	None	CCR5
SE600213	None	None	None	None	None	None	None	None	None	None	CCR5
SE600314	None	**L10IL**	None	E138A	None	None	None	E138A	None	None	CCR5
SE600415	None	None	None	None	None	None	None	**N348I**	None	None	CCR5
SE600122	None	V11I	None	None	None	V11I	None	None	None	None	CCR5
SE600516	None	None	None	None	None	None	None	None	None	None	CCR5
SE601017	None	None	None	None	None	None	None	None	None	L74I	CXCR4
SE601018	None	None	None	E138A	None	None	None	E138A	None	None	CCR5
SE601021	None	None	None	None	None	None	None	None	None	None	CXCR4
SE602019	None	L10I, K20I	None	None	None	L10I, K20I	None	None	None	None	CCR5
SE602020	None	K20I	M184V	V106A, F227L, **Y318FY**	None	K20I	M184V	V106A, F227L	None	None	CXCR4
SE602024	None	K20I	None	None	None	K20I	None	None	None	None	CCR5

The mismatches are marked in bold.

## Discussion

In the present study, we have developed a simple, labour and time-efficient HIV-NFLG sequencing protocol that can be used for large-scale molecular epidemiological studies. This assay can identify the phylogenetic transmission cluster in a local HIV-1 epidemic. Therefore, it has a potential role in the prevention of HIV-1 transmission in the high-risk groups from a public health perspective. Moreover, the assay extends the genotypic GRT to cover all HIV-1 drug target sites (PR inhibitors, RTIs, IN and fusion inhibitors). It also includes genotypic co-receptor tropism analysis and genetic analysis of the non-drug target sites that potentially affect the drug efficiency as well as resistance (e.g. Gag).

Molecular epidemiological studies often use smaller gene fragments that can potentially underestimate the event of recombination. Our earlier study showed significant increase in recombinants when two or more genes from different parts of the HIV-1 genome (e.g. *gag, pol* and *env*) were used [4]. Therefore, the use of NFLG is ideal to be used as a tool for HIV-1 subtyping [34]. It also enables an understanding of the dynamics of the HIV pandemic at a population level [3]. Earlier studies that developed full-length genome are either expensive, time consuming or hampered by low throughput that is often restricted to one HIV-1 subtype [2]. A HIV-NFLG sequencing protocol from plasma viral RNA has been developed using two or three fragments to cover 9 kb genome [17]. However, the method was limited by its efficiency in samples having a viral load <10,000 copies/ml and a subtype-independent applicability was not mentioned. If a high-quality RNA sample is provided, NFLG sequences from patients with viral loads as low as 3000 copies/ml can be obtained, which is a great advantage of the presented assay compared to the earlier method [17].

Most importantly, the current method can detect four major subtypes and recombinant forms (HIV-1B, HIV-1C, 01_AE and 02_AG), which are responsible for >80% of global infections when combined [19]. The method can be potentially applied to all major pure and recombinant HIV-1 strains as the primers applied here had >95% sequence identity to them. Recently, a universal amplification protocol has been developed that amplifies HIV-1 genome in four PCR fragments followed by sequencing in a next generation sequencing (NGS) platform [3]. However, the method is more labour-intensive and the application of the method is limited in low- and middle-income countries (LMICs) due to limited availability of NGS and corresponding experts to run the assays. The calculated costs for NGS strongly depend on the number of samples processed and increase if only a few numbers of samples have to be analyzed. In our current NFLG method, the cost per sample is between $130 and $140 and even one sample can be run without any change in effective cost. The overall time taken for the method is five days.

The current method can also be applied for genotypic resistance testing as it showed high concordance in determining the DRM compared to gold standard ViroSeq™ System. Additionally, it can determine clinically important N348I that confers resistance to RTI [8]. Recently, a single assay for HIV-1 GRT and co-receptor tropism assay, based on deep sequencing, has been developed [35]. Though the assay is high throughput, the application of the method is limited in LMICs, due to limited availability of NGS in those settings. However, with this current method, in a single assay it is now possible to determine PI, RTI and INI resistance mutations and co-receptor tropism in LMICs also. Finally, Gag has shown to act synergistically to confer resistance to PIs [9,10]. The insertions in Gag-p6 have recently shown to be associated with PI-therapy failure in Indian HIV-1C viruses [36]. The current method is also helpful in determining the full Gag including p6 region.

Our study has certain technical aspects that merit comments. First, the quality of RNA is important for successful amplification. Therefore, highly lipemic or lysed samples should not be used. Second, the sequencing of the Env region may require more primers [22]. This is due to higher occurrence of poly-A stretches in the *env* variable regions (V1 to V5; more specifically in the V4 region), which can suddenly stop the sequencing reaction. Third, in two positions mixed populations were detected by ViroSeq™ but not by NFLG. This kind of results was also noted in earlier studies [37,38] and might be due to the variant calling. The method is less efficient in samples with viral load <3000 copies/ml. Furthermore, extensive mutations in the primer binding sites can result in failure of amplification as observed in most HIV-diagnostic assays. However, a major merit of this assay is its subtype independency. In the current study, the samples were from patients who got HIV infected in different countries – Sweden, Zimbabwe, Ethiopia, Thailand, Senegal, Cameroon and Eritrea – with four major subtypes. Another important factor is the use of this method in clinical management of drug resistance. The input plasma sample volume is only 140 µl. Thus, no additional blood sample is required and the assay can be merged with routine viral load and CD4 testing.

## Conclusions

In conclusion, we demonstrated a subtype-independent HIV-NFLG sequencing method that is a simple, cost and labour-efficient and promising approach. The method can be used in large-scale population-based molecular epidemiological studies that can identify the HIV-1 recombinant forms more accurately as well as population dynamics of HIV-1 spread. It can also be used in extended genotypic resistance testing that can evaluate DRMs in PR, RT and IN genes. Genotypic co-receptor tropism for evaluation of co-receptor antagonist usage can also be done in one single assay. Therefore, the application of this method in clinical care can improve patient management strategies through the accurate identification the strains. A better understanding of the population dynamics of HIV-1 within pandemics could enable the development of an effective intervention for control and prevention.

## Supplementary Material

Subtype-independent near full-length HIV-1 genome sequencing and assembly to be used in large molecular epidemiological studies and clinical management
