# Supplementary material for: Subtype-independent near full-length HIV-1 genome sequencing and assembly to be used in large molecular epidemiological studies and clinical management
Source: J Int AIDS Soc. 2015 Jun 25;18(1):20035. doi: 10.7448/IAS.18.1.20035 (PMC4482814; doi:10.7448/IAS.18.1.20035)
Supplement: Subtype-independent near full-length HIV-1 genome sequencing and assembly to be used in large molecular epidemiological studies and clinical management [file JIAS-18-20035-s001.pdf]

```
#!/usr/bin/perl
use strict;
use warnings;
$|=1

##### General Information ######
#Use multiple alignment in FASTA format as input. Choose txt file location for output.
#First sequence has to be reference every other sequence is compared to.

#Coordinates for HIV-1 gene map can be derived from SequenceLocator at
[http://www.hiv.lanl.gov/content/sequence/LOCATE/locate.html]
#using the reference sequence
#Create gene map:
#Frame1 Gag, Vif, Tat_Exon2, Nef
#Frame2 Tat_Exon1, Vpu, Rev_Exon2
#Frame3 Pol, Vpr, Rev_Exon1, Env
#my $gag = 1;
#my $pol = 2;
#my $vif = 3;
#my $vpr = 4;
#my $tat = 5;
#my $rev = 6;
#my $vpu = 7;
#my $env = 8;
#my $nef = 9;
#print OUT "$gag " x ."$same "x ."$vif" x ."$same "x ."$tat "x ."$same "x ."$nef "x ."\n";
#print OUT "$same "x ."$tat "x ."$same "x ."$vpu "x ."$same "x ."$rev "x ."$same "x ."\n";
#print OUT "$same "x ."$pol "x ."$same "x ."$vpr "x ."$same "x ."$rev "x ."$same "x ."$env "x ."$same "x ."\n";
########################

#Infile and Outfile
my $infile = $ARGV[0];
open (IN,$infile) or die "Cannot find txt file with protein alignments\n";
my $outfile = $ARGV[1];
open (OUT,'>'.$outfile) or die "No outputfile declared or no rights to write\n";

#Variables for color coding
my $same = 0;
my $del = 10;
my $ins = 11;
my $mut = 12;

#Variables for genome comparison
my $refheader = <IN>;
chomp $refheader;
my $refseq = <IN>;
chomp $refseq;
my @refary = split(//,$refseq);
my $leng = scalar @refary;
my @mutary;
```

```
while(<IN>){
	my $line = $_;
	chomp $line;
	if($line =~ />/){
		next;
	}else{
		@mutary = split(//,$line);
		for(my $i = 0; $i < $leng; $i++){
			#Match in Ref and Sequence => 0
			if($mutary[$i] eq $refary[$i]){
				print OUT "$same ";
			#Not same but gap in Sequence => Deletion => 10
			}elsif($mutary[$i] eq "-"){
				print OUT "$del ";
			#Not same but gap in Ref => Insertion => 11
			}elsif($refary[$i] eq "-"){
				print OUT "$ins ";
			#Not same but no gaps => Mutation => 12
			}else{
				print OUT "$mut ";
			}
		}
	print OUT "\n";
	}
}

exit;
```
